# Supplementary material for: Taxonomy Complexity of Some Tyrrhenian Endemic Limonium Species Belonging to L. multiforme Group (Plumbaginaceae): New Insights from Molecular and Morphometric Analyses
Source: Plants (Basel). 2022 Nov 18;11(22):3163. doi: 10.3390/plants11223163 (PMC9693374; doi:10.3390/plants11223163)
Supplement: Supplementary file 1 [file plants-11-03163-s001.zip › plants-1957475-supplementary/SupMat_Files S1-S3_Duilio et al_Limonium/Supplementary Material File S3_CP_ML_tree.pdf]

Supplementary Material for

Taxonomy Complexity of Some Tyrrhenian Endemic *Limonium* Species Belonging to *L. multiforme* Group (Plumbaginaceae): New Insights from Molecular and Morphometric Analyses.

Duilio Iamonico, Olga De Castro, Emanuela Di Iorio, Gianluca Nicolella and Mauro Iberite

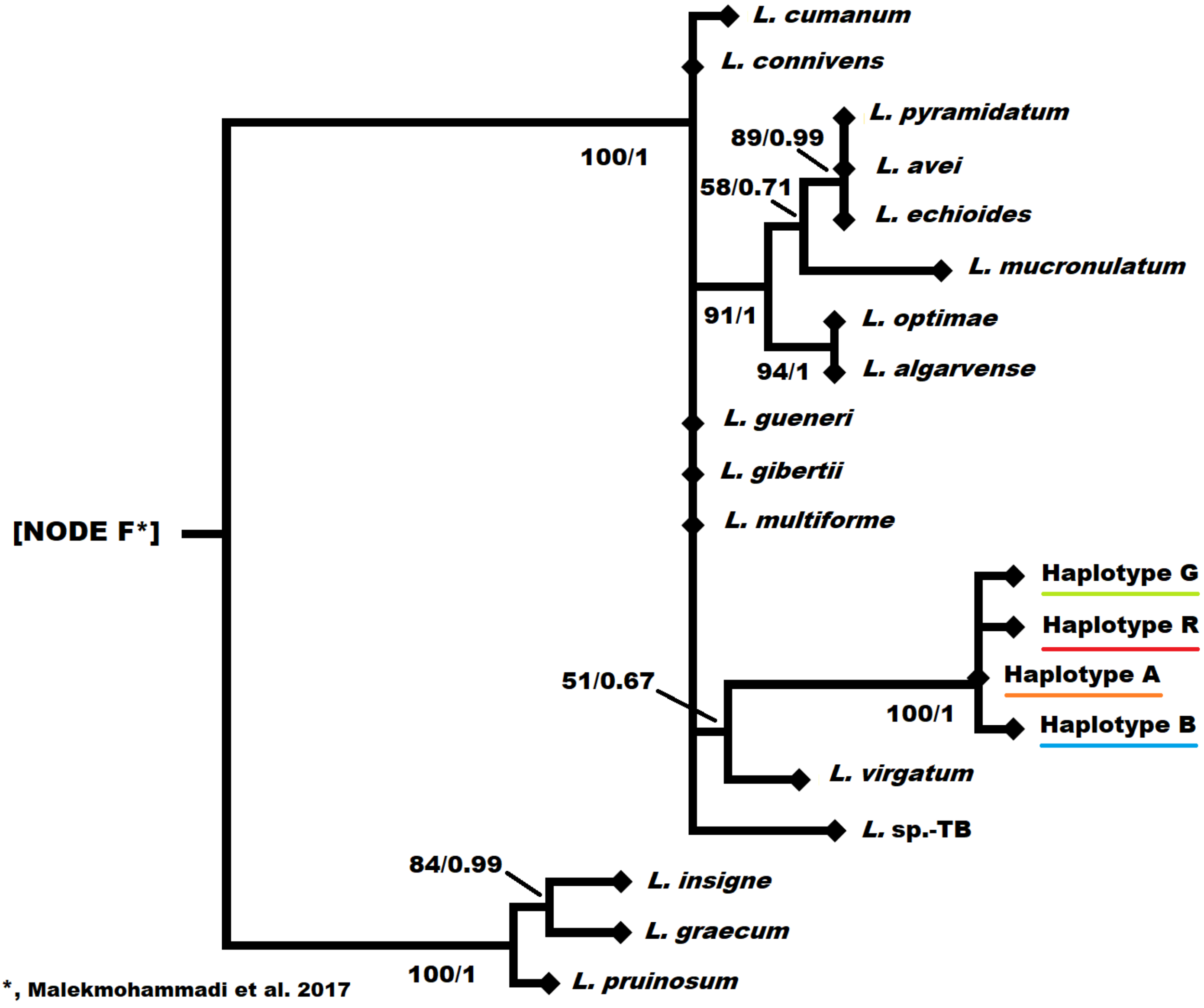

Supplementary Material S3. Detail of maximum likelihood (ML) phylogram of plastid sequences [*pet*B-*pet*D IGS+petD intron and *trn*L$^{(UAA)}$-*trn*G$^{(GAA)}$ IGS] regarding "*Limonium graecum* clade" which corresponds to the node F of Figure 2 of Malekmohammadi et al. 2017 [4]. Same topology was observed in Bayesian Inference (BI). ML bootstrap values followed by bayesian posterior probabilities are shown below the branches (values > 50%).
